# Prognostic impact of tumor size on patients with metastatic colorectal cancer: a large SEER-based retrospective cohort study

**DOI:** 10.1007/s13304-023-01533-4

**Published:** 2023-05-18

**Authors:** Qi Zhang, Baosong Li, Shiyao Zhang, Qianpeng Huang, Maorun Zhang, Gang Liu

**Affiliations:** 1 Department of General Surgery, Tianjin Medical University General Hospital, Tianjin, 300052 China; 2 Department of Colorectal Surgery, Binzhou Medical University Hospital, Binzhou, 256603 China

**Keywords:** Metastatic colorectal cancer, SEER, Tumor size, Prognostic model

## Abstract

Given the poor prognosis of metastatic colorectal cancer (mCRC), this research aimed to investigate the correlation between tumor size and prognosis, and develop a novel prediction model to guide individualized treatment. Patients pathologically diagnosed with mCRC were recruited from the Surveillance, Epidemiology, and End Results (SEER) database between 2010 and 2015, and were randomly divided (7:3 ratio) into a training cohort (*n* = 5597) and a validation cohort (*n* = 2398). Kaplan–Meier curves were used to analyze the relationship between tumor size and overall survival (OS). Univariate Cox analysis was applied to assess the factors associated with the prognosis of mCRC patients in the training cohort, and then multivariate Cox analysis was used to construct a nomogram model. The area under the receiver-operating characteristics curve (AUC) and calibration curve were used to evaluate the predictive ability of the model. Patients with larger tumors had a worse prognosis. While brain metastases were associated with larger tumors compared to liver or lung metastases, bone metastases tended to be associated with smaller tumors. Multivariate Cox analysis revealed that tumor size was an independent prognostic risk factor (HR 1.28, 95% CI 1.19–1.38), in addition to the other ten variables (age, race, primary site, grade, histology, T stage, N stage, chemotherapy, CEA level and metastases site). The 1-, 3-, and 5-year OS nomogram model yielded AUC values of more than 0.70 in both the training and validation cohorts, and its predictive performance was superior to that of the traditional TNM stage. Calibration plots demonstrated a good agreement between the predicted and observed 1-, 3-, and 5-year OS outcomes in both cohorts. The size of primary tumor was found to be significantly associated with prognosis of mCRC, and was also correlated with specific metastatic organ. In this study, we presented the first effort to create and validate a novel nomogram for predicting 1-, 3- and 5-year OS probabilities of mCRC. The prognostic nomogram was demonstrated to have an excellent predictive ability in estimating individualized OS of patients with mCRC.

## Introduction

Among all malignant tumors, colorectal cancer (CRC) has the third-highest incidence rate and the second-highest mortality rate [1]. Distant metastasis is the main cause of death for those affected by CRC and, currently, around half of all CRC patients are diagnosed at stage IV (metastatic colorectal cancer, mCRC) [2, 3]. Subsequently, considerable financial investment has been made to develop treatments that can both screen for and reduce the incidence of cancer mortality [4]. Notably, mCRC has a better prognosis than other gastrointestinal metastatic cancers [1]. However, CRC is a heterogeneous disease and prognosis varies between patients, so the development of prognostic risk models could further improve treatment strategies for stratified management. Most prognostic risk models currently available have been built using data from the whole CRC population or from those with postoperative stage II and stage III CRC [5–7], thus a new prediction model for mCRC is required.

The American Joint Committee on Cancer (AJCC) system of tumor (T), nodes (N), and metastases (M) is a widely accepted approach for classifying cancer risk in patients with colorectal cancer [8]. Resected specimens are examined for a pathological stage (pTNM), while radiographic and endoscopic examinations are used to assign a clinical stage (cTNM). In addition, a post-neoadjuvant pathological stage (ypTNM) is employed to further stratify patients who have had systemic or radiation treatment prior to surgery for a colorectal primary tumor. Given its substantial influence on prognosis, recurrence, survival, and clinical management, the size of solid tumors in the TNM staging system is considered a valid indicator of its prognostic relevance [9]; however, there is debate as to its efficacy in assessing the risk of colorectal cancer [10, 11]. In addition to spreading horizontally, CRC can also invade deeply into the layers of the colon wall. The depth of tumor invasion, rather than tumor size, is employed in the current AJCC colorectal cancer T stage. Retrospective studies have also shown a direct link between tumor size or maximum horizontal tumor diameter and improved survival in CRC patients [12–16].

Even though the majority of these studies employed small sample sizes, further research is necessary owing to the potential for tumor size to serve as an indicator of CRC survival. This study employed the largest cohort to date to explore the effect of tumor size on mCRC patients.

## Materials and methods

### Patients’ selection

After being granted access to study data files with the reference number 12271-Nov2019, information on patients with newly diagnosed CRC from 2010 to 2015 was taken from the Surveillance, Epidemiology and End Results (SEER) database. The SEER data collection (https://seer.cancer.gov/seerstat/), sponsored by the National Cancer Institute, contains information on the incidence and survival characteristics of malignancies among 26% of the population and 18 cancer registries in the USA.

Overall survival (OS) information was also collected, together with clinicopathological characteristics and pertinent therapy details. The following criteria were met by the patients: (1) patients had to be between the ages of 18 and 75; (2) they had to have stage IV CRC pathologically determined; and (3) CRC was the only primary cancer. Patients with insufficient staging, missing metastatic information, diagnoses made only through autopsy, and patients whose prognosis was unknown were also disqualified. In addition, the tumor size indicated in this study was the primary tumor size.

### Statistical analysis

X-tile 3.6.1, SPSS 26.0, and R 4.1.1 were the application software used in this study’s statistical analysis. Baseline characteristics were shown. Categorical data were given as frequencies with percentages, while continuous variables were expressed as the median [IQR]. Categorical data were compared using a *χ*^2^ test. The Cox regression model was used to analyze the survival difference, and then a nomogram model was created using the results of cox regression. The nomogram’s discrimination power was confirmed by the receiver-operator characteristic (ROC) curve, which also exhibited prediction and decision curve analysis (DCA) of nomogram for predicting patients’ OS at 1, 3, and 5 years. All statistical analyses were conducted bilaterally, and a *P* value of 0.05 or less was regarded as statistically significant.

## Results

### Baseline demographic and clinicopathological characteristics

**Fig. 1:**
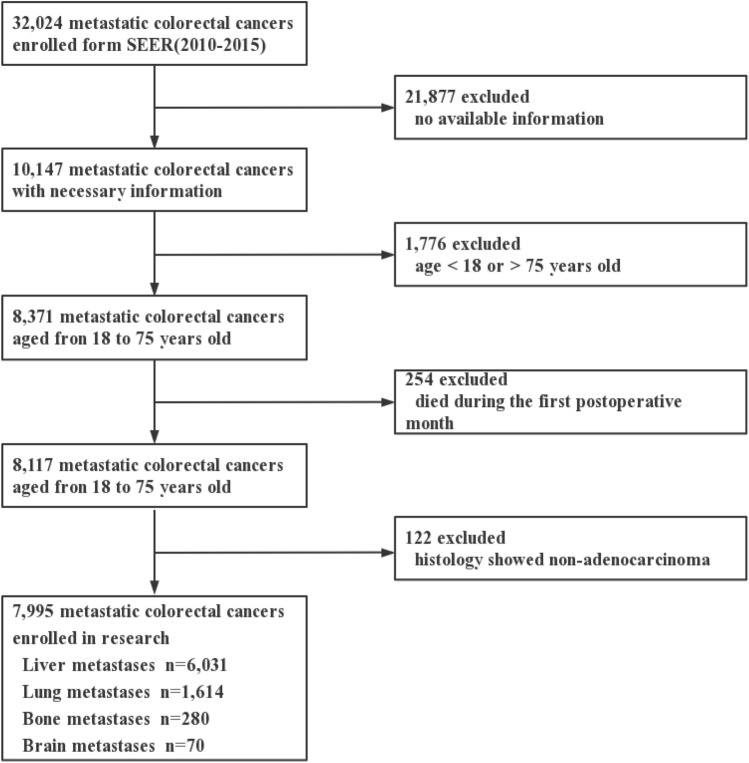
Flow chart of patients’ selection from SEER database. *SEER* Surveillance, Epidemiology, and End Results

The baseline demographic and clinicopathological characteristics of the study population are shown in Table 1. A total of 7995 patients were included in our cohort (Fig. 1). The median age of these patients was 57.63 (IQR 54.42–60.84). 58.5% (*n* = 5268) were male, and nearly third of fourths were white (*n* = 5982, 74.8%). More patients had tumor in left colon (*n* = 3132, 39.2%), while others were in right colon (*n* = 1417, 17.7%), transverse colon (*n* = 590, 7.4%) and rectum (*n* = 2856, 35.7%). In this cohort, 72.6% (*n* = 5801) were histopathologically moderately differentiated and 94.6% (*n* = 7567) were adenocarcinoma. Radiation was given to 16.5% (*n* = 1321) of all patients, and 83.2% (*n* = 6652) of all patients accepted chemotherapy. 82.4% (*n* = 6591) of all patients showed CEA-positive results. Among all patients with distant metastases, 75.4% (*n* = 6031) of patients had liver metastasis, 20.2% (*n* = 1614) of patients had lung metastases, 3.5% (*n* = 280) had bone metastases, and 0.9% (*n* = 70) had brain metastases. The training cohort and validation cohort were then divided at a ratio of 7:3, and as there were no statistically significant differences between the 2 patient groups (*P* > 0.05), they were considered comparable (Table 2).

**Table 1:** Baseline clinicopathologic characteristics of mCRC patients with different metastases

Variables	Total (*n* = 7995)	No. of patients (%)											
		Liver metastases			Lung metastases			Bone metastases			Brain metastases		
		Yes (*n* = 6031)	No (*n* = 1964)	*P* value	Yes (*n* = 1614)	No (*n* = 6381)	*P* value	Yes (*n* = 280)	No (*n* = 7715)	*P* value	Yes (*n* = 70)	No (*n* = 7925)	*P* value
Age				0.047*			0.019*			0.437			0.559
≤ 60	4502 (56.3)	3434 (56.9)	1068 (54.4)		867 (53.7)	3635 (57.0)		164 (58.6)	4338 (56.2)		37 (52.9)	4465 (56.3)	
> 60	3493 (43.7)	2597 (43.1)	896 (45.6)		747 (46.3)	2746 (43.0)		116 (41.4)	3377 (43.8)		33 (47.1)	3460 (43.7)	
Sex				0.008*			0.003*			0.446			0.335
Female	3317 (41.5)	2452 (40.7)	865 (44.0)		722 (44.7)	2595 (40.7)		110 (39.3)	3207 (41.6)		33 (47.1)	3284 (41.4)	
Male	4678 (58.5)	3579 (59.3)	1099 (56.0)		892 (55.3)	3786 (59.3)		170 (60.7)	4508 (58.4)		37 (52.9)	4641 (58.6)	
Race				0.133			0.034*			0.938			0.360
White	5982 (74.8)	4542 (75.3)	1440 (73.3)		1172 (72.6)	4810 (75.4)		212 (75.7)	5770 (74.8)		56 (80.0)	5926 (74.8)	
Black	1137 (14.2)	848 (14.1)	286 (14.6)		238 (14.7)	896 (14.0)		38 (13.6)	1096 (14.2)		10 (14.3)	1124 (14.2)	
Other	879 (11.0)	641 (10.6)	238 (12.1)		204 (12.7)	675 (10.6)		30 (10.7)	849 (11.0)		4 (5.7)	875 (11.0)	
Primary site				< 0.001*			< 0.001*			0.002*			0.007*
Right colon	1417 (17.7)	1105 (18.3)	312 (15.9)		242 (15.0)	1175 (18.4)		48 (17.2)	1369 (17.7)		22 (31.4)	1395 (17.6)	
Transverse colon	590 (7.4)	458 (7.6)	132 (6.7)		109 (6.8)	481 (7.5)		21 (7.5)	569 (7.4)		2 (2.9)	588 (7.4)	
Left colon	3132 (39.2)	2474 (41.0)	658 (33.5)		556 (34.4)	2576 (40.4)		83 (29.6)	3049 (39.5)		19 (27.1)	3113 (39.3)	
Rectum	2856 (35.7)	1994 (33.1)	862 (43.9)		707 (43.8)	2149 (33.7)		128 (45.7)	2728 (35.4)		27 (38.6)	2829 (35.7)	
Tumor size (cm)	5.60	5.55	5.76	0.004*	5.76	5.56	0.038*	5.75	5.59	0.128	5.87	5.60	0.171
Grade				0.308			0.222			< 0.001*			0.161
I	361 (4.5)	263 (4.3)	98 (5.0)		85 (5.3)	276 (4.3)		9 (3.2)	352 (4.6)		4 (5.7)	357 (4.5)	
II	5801 (72.6)	4400 (73.0)	1401 (71.3)		1180 (73.1)	4621 (72.4)		174 (62.1)	5627 (72.9)		47 (67.1)	5754 (72.6)	
III	1553 (19.4)	1152 (19.1)	401 (20.4)		299 (18.5)	1254 (19.7)		83 (29.7)	1470 (19.1)		19 (27.2)	1534 (19.4)	
IV	280 (3.5)	216 (3.6)	64 (3.3)		50 (3.1)	230 (3.6)		14 (5.0)	266 (3.4)		0 (0.0)	280 (3.5)	
Histology				0.741			0.941			0.278			0.351
Mucinous adenocarcinoma	428 (5.4)	320 (5.3)	108 (5.5)		87 (5.4)	341 (5.3)		19 (6.8)	409 (5.3)		2 (2.9)	426 (5.4)	
Adenocarcinoma	7567 (94.6)	5711 (94.7)	1856 (94.5)		1527 (94.6)	6040 (94.7)		261 (93.2)	7306 (94.7)		68 (97.1)	7499 (94.6)	
AJCC T stage				< 0.001*			< 0.001*			< 0.001*			0.562
T1	682 (8.6)	465 (7.7)	217 (11.0)		171 (10.6)	511 (8.0)		37 (13.2)	645 (8.3)		9 (12.9)	673 (8.5)	
T2	273 (3.4)	205 (3.4)	68 (3.5)		55 (3.4)	218 (3.4)		10 (3.6)	263 (3.4)		3 (4.2)	270 (3.4)	
T3	4449 (55.6)	3456 (57.3)	993 (50.5)		831 (51.5)	3618 (56.7)		124 (44.3)	4325 (56.1)		38 (57.3)	4411 (55.7)	
T4	2591 (32.4)	1905 (31.6)	686 (35.0)		557 (34.5)	2034 (31.9)		109 (38.9)	2482 (32.2)		20 (28.6)	2571 (32.4)	
AJCC N stage				0.234			0.044*			0.767			0.021*
NO	1856 (23.2)	1378 (22.8)	478 (24.3)		401 (24.8)	1455 (22.8)		60 (21.4)	1796 (23.3)		17 (24.3)	1839 (23.2)	
N1	3370 (42.2)	2537 (42.1)	833 (42.4)		694 (43.0)	2676 (41.9)		120 (42.9)	3250 (42.1)		19 (27.1)	3351 (42.3)	
N2	2769 (34.6)	2116 (35.1)	653 (33.3)		519 (32.2)	2250 (35.3)		100 (35.7)	2669 (34.6)		34 (48.6)	2735 (34.5)	
Radiation				< 0.001*			< 0.001*			< 0.001*			<0.001*
No/unknown	6674 (83.5)	5199 (86.2)	1475 (75.1)		1278 (79.2)	5396 (84.6)		172 (61.4)	6502 (84.3)		25 (35.7)	6649 (83.9)	
Yes	1321 (16.5)	832 (13.8)	489 (24.9)		336 (20.8)	985 (15.4)		108 (38.6)	1213 (15.7)		45 (64.3)	1276 (16.1)	
Chemotherapy				0.008*			0.075			0.749			< 0.001*
No/unknown	1343 (16.8)	975 (16.2)	368 (18.7)		295 (18.3)	1048 (16.4)		49 (17.5)	1294 (16.8)		24 (34.3)	1319 (16.6)	
Yes	6652 (83.2)	5056 (83.8)	1596 (81.3)		1319 (81.7)	5333 (83.6)		231 (82.5)	6421 (83.2)		46 (65.7)	6606 (83.4)	
CEA level				0.687			0.291			0.729			< 0.001*
Negative	1404 (17.6)	1065 (17.7)	339 (17.3)		269 (16.7)	1135 (17.8)		47 (16.8)	1357 (17.6)		23 (32.9)	1381 (17.4)	
Positive	6591 (82.4)	4966 (82.3)	1625 (82.7)		1345 (83.3)	5246 (82.2)		233 (83.2)	6358 (82.4)		47 (67.1)	6544 (82.6)

**Table 2:** Clinicopathologic characteristics of training cohort (n = 5597) and validation cohort (n = 2398)

Variables	No. of patients (%)		*P* value
	Training cohort (*n* = 5597)	Validation cohort (*n* = 2398)	
Age			0.151
≤ 60	3122 (55.8%)	1380 (57.5%)	
> 60	2475 (44.2%)	1018 (42.5%)	
Sex			0.469
Female	3290 (58.8%)	1388 (57.9%)	
Male	2307 (41.2%)	1010 (42.1%)	
Race			0.547
White	4189 (74.8%)	1793 (74.8%)	
Black	782 (14.0%)	352 (14.7%)	
Other	626 (11.2%)	253 (10.6%)	
Primary site			0.734
Right colon	982 (17.5%)	435 (18.1%)	
Transverse colon	417 (7.5%)	173 (7.2%)	
Left colon	2180 (38.9%)	952 (39.7%)	
Rectum	2018 (36.1%)	838 (34.9%)	
Tumor size (cm)			0.221
< 4.6	2279 (40.7%)	927 (38.7%)	
4.6–6.9	1985 (35.5%)	885 (36.9%)	
> 6.9	1333 (23.8%)	586 (24.4%)	
Grade			0.592
I	259 (4.6%)	102 (4.3%)	
II	4063 (72.6%)	1738 (72.5%)	
III	1088 (19.4%)	465 (19.4%)	
IV	187 (3.3%)	93 (3.9%)	
Histology			0.378
Mucinous adenocarcinoma	309 (5.5%)	119 (5.0%)	
Adenocarcinoma	5288 (94.5%)	2279 (95.0%)	
AJCC T stage			0.680
T1	491 (8.8%)	191 (8.0%)	
T2	193 (3.4%)	80 (3.3%)	
T3	3104 (55.5%)	1345 (56.1%)	
T4	1809 (32.3%)	782 (32.6%)	
AJCC N stage			0.835
NO	1289 (23.0%)	567 (23.6%)	
N1	2366 (42.3%)	1004 (41.9%)	
N2	1942 (34.7%)	827 (34.5%)	
Radiation			0.757
No/unknown	4667 (83.4%)	2007 (83.7%)	
Yes	930 (16.6%)	391 (16.3%)	
Chemotherapy			0.571
No/unknown	931 (16.6%)	412 (17.2%)	
Yes	4666 (83.4%)	1986 (82.8%)	
CEA level			0.390
Negative	969 (17.3%)	435 (18.1%)	
Positive	4628 (82.7%)	1963 (81.9%)	
Metastases site			0.239
Liver	4223 (75.5%)	1808 (75.4%)	
Lung	1125 (20.1%)	489 (20.4%)	
Bone	206 (3.7%)	74 (3.1%)	
Brain	43 (0.8%)	27 (1.1%)	

### Metastasis characteristics based on different tumor sizes and metastasis sites

**Fig. 2:**
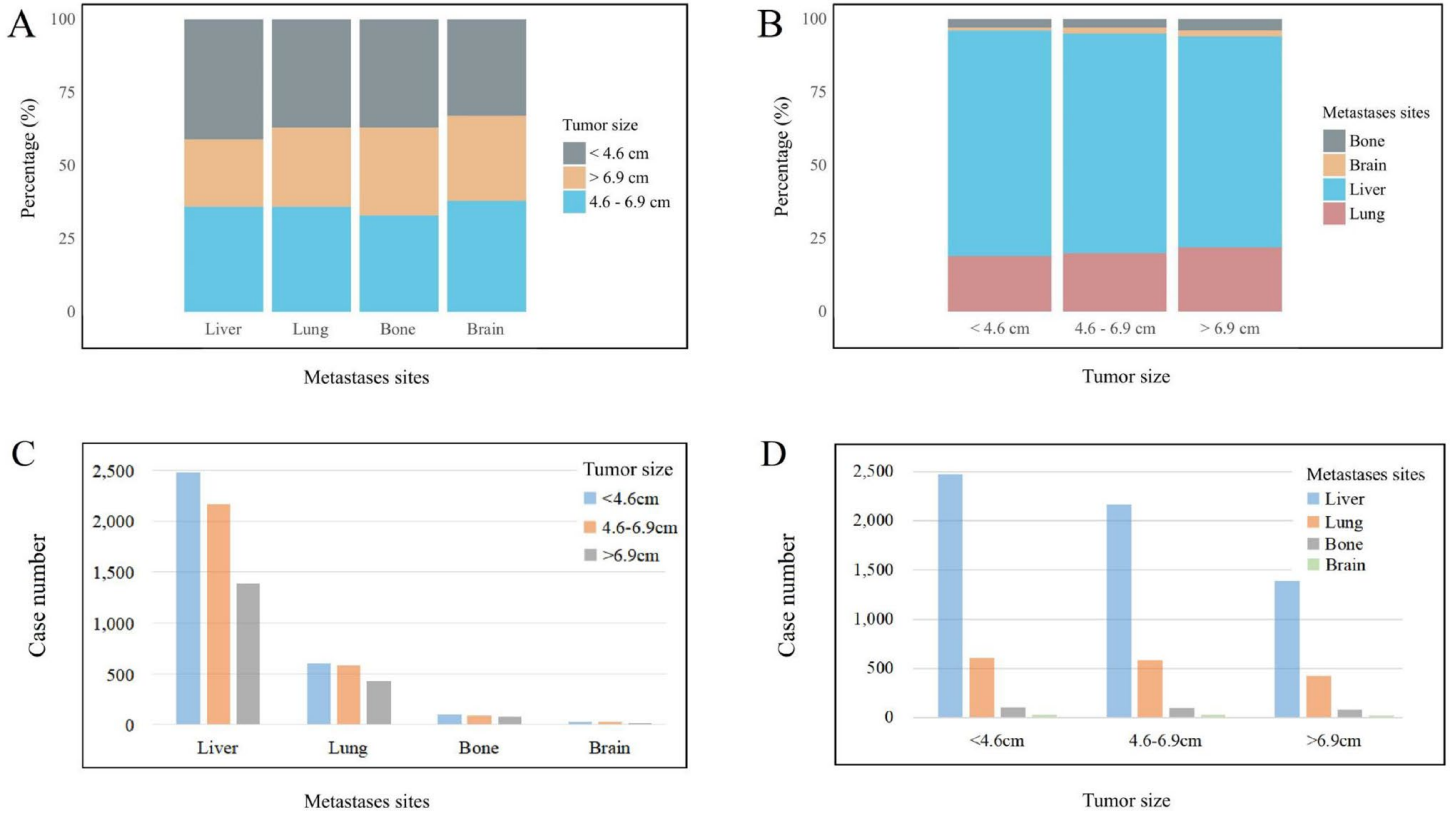
Percentage and cases of each distant metastases site. **A**, **C** Different tumor size based on different metastases sites. **B**, **D** Different metastases sites based on different tumor size

**Fig. 3:**
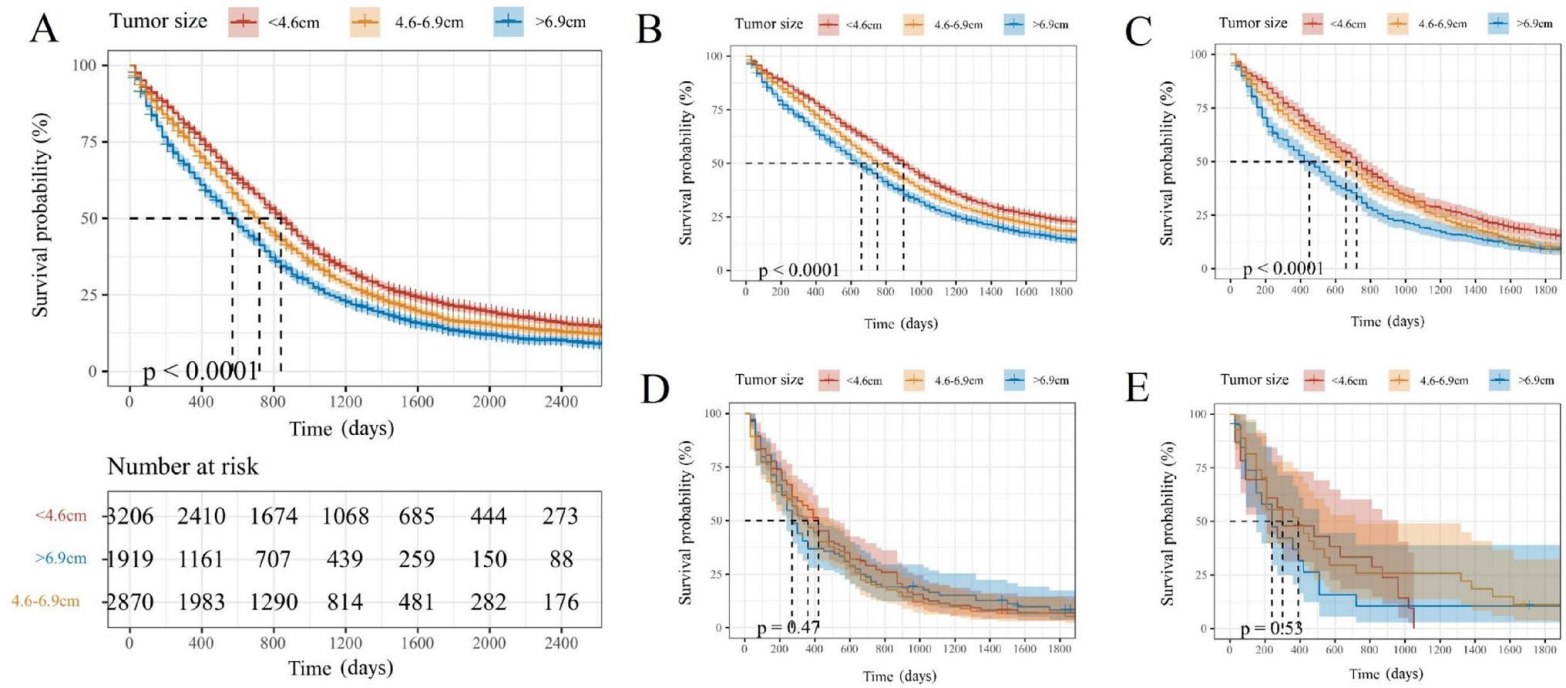
Kaplan–Meier survival curves drawn by different cutoff value calculated by X-tile of all mCRC patients (**A**) and patients with liver metastases (**B**), lung metastases (**C**), bone metastases (**D**) and brain metastases (**E**)

The best cutoff value calculated by X-tile was < 4.6 cm, 4.6–6.9 cm and > 6.9 cm, as shown in Fig. 2A; liver metastases were the most common metastatic site among the three groups. As the tumor size was large, the proportion of bone metastases and brain metastases increased. Brain metastases were the least frequent metastatic pattern among tumor size groups. As shown in Fig. 2B, in the liver, lung and bone metastases group, the proportion with tumor size < 4.6 cm was obviously higher than those with tumor size ≥ 4.6 cm while brain metastases group was more likely have a primary tumor size between 4.6 and 6.9 cm. The results showed that the tumor size of liver metastases and lung metastases was shown in the relatively similar tumor size, that the bone metastases had the smallest tumor size, and that the brain metastases had the largest tumor size after using x-tile to look for the most effective cutoff value of tumor size in specific metastases. The K–M curves showed that the patients’ prognosis was worse as tumor size increased (Fig. 3).

### Prognostic factors affecting the OS of patients

According to univariate Cox analysis (Table 3), age, race, primary site, tumor size, grade, histology, T stage, N stage, radiation, chemotherapy, CEA level and metastases site were prognostic factors affecting OS in training cohort (all *P* < 0.05). The results of multivariate Cox analysis showed that radiation was not an independent prognostic factor while other 11 factors were effective (all *P* < 0.05).

**Table 3:** Univariate and multivariate Cox regression analyses for overall survival in training cohort

Variables	Univariate analysis			Multivariate analysis		
	HR	95% CI	*P* value	HR	95% CI	*P* value
Age						
≤ 60	Reference	Reference		Reference	Reference	
> 60	1.319	1.245–1.396	< 0.001*	1.224	1.154–1.298	< 0.001*
Sex						
Female	Reference	Reference				
Male	0.951	0.897–1.008	0.091			
Race						
White	Reference	Reference		Reference	Reference	
Black	1.243	1.147–1.348	< 0.001*	1.237	1.165–1.389	< 0.001*
Other	1.032	0.941–1.132	0.501	1.005	0.935–1.144	0.924
Primary site						
Right colon	Reference	Reference		Reference	Reference	
Transverse colon	0.865	0.765–0.978	0.021*	0.922	0.815–1.043	0.196
Left colon	0.697	0.643–0.756	< 0.001*	0.772	0.711–0.839	< 0.001*
Rectum	0.701	0.646–0.761	< 0.001*	0.859	0.786–0.940	0.021*
Tumor size (cm)						
< 4.6	Reference	Reference		Reference	Reference	
4.6–6.9	1.153	1.079–1.231	< 0.001*	1.072	1.003–1.146	0.040*
> 6.9	1.396	1.298–1.502	< 0.001*	1.281	1.189–1.380	< 0.001*
Grade						
I	Reference	Reference		Reference	Reference	
II	0.985	0.856–1.134	0.993	1.002	0.861–1.167	0.834
III	1.421	1.222–1.652	< 0.001*	1.430	1.215–1.684	< 0.001*
IV	1.523	1.243–1.867	< 0.001*	1.542	1.238–1.919	< 0.001*
Histology						
Mucinous adenocarcinoma	Reference	Reference		Reference	Reference	
Adenocarcinoma	0.770	0.681–0.870	< 0.001*	0.868	0.767–0.982	0.025*
AJCC T stage						
T1	Reference	Reference		Reference	Reference	
T2	0.488	0.403–0.591	< 0.001*	0.466	0.384–0.565	< 0.001*
T3	0.625	0.565–0.692	< 0.001*	0.554	0.499–0.615	< 0.001*
T4	0.985	0.887–1.093	0.772	0.785	0.703–0.877	< 0.001*
AJCC N stage						
NO	Reference	Reference		Reference	Reference	
N1	1.128	1.046–1.216	0.002*	1.173	1.086–1.267	< 0.001*
N2	1.405	1.300–1.518	< 0.001*	1.444	1.331–1.567	< 0.001*
Radiation						
No/unknown	Reference	Reference		Reference	Reference	
Yes	0.841	0.778–0.909	< 0.001*	0.976	0.893–1.067	0.588
Chemotherapy						
No/unknown	Reference	Reference		Reference	Reference	
Yes	0.404	0.375–0.435	< 0.001*	0.418	0.387–0.451	< 0.001*
CEA level						
Negative	Reference	Reference		Reference	Reference	
Positive	1.632	1.506–1.768	< 0.001*	1.680	1.549–1.821	< 0.001*
Metastases site						
Liver	Reference	Reference		Reference	Reference	
Lung	1.318	1.228–1.413	< 0.001*	1.294	1.206–1.390	< 0.001*
Bone	2.156	1.863–2.495	< 0.001*	1.934	1.669–2.242	< 0.001*
Brain	1.728	1.261–2.370	0.001*	1.350	0.980–1.860	0.067

### Construction of nomogram prediction model

**Fig. 4:**
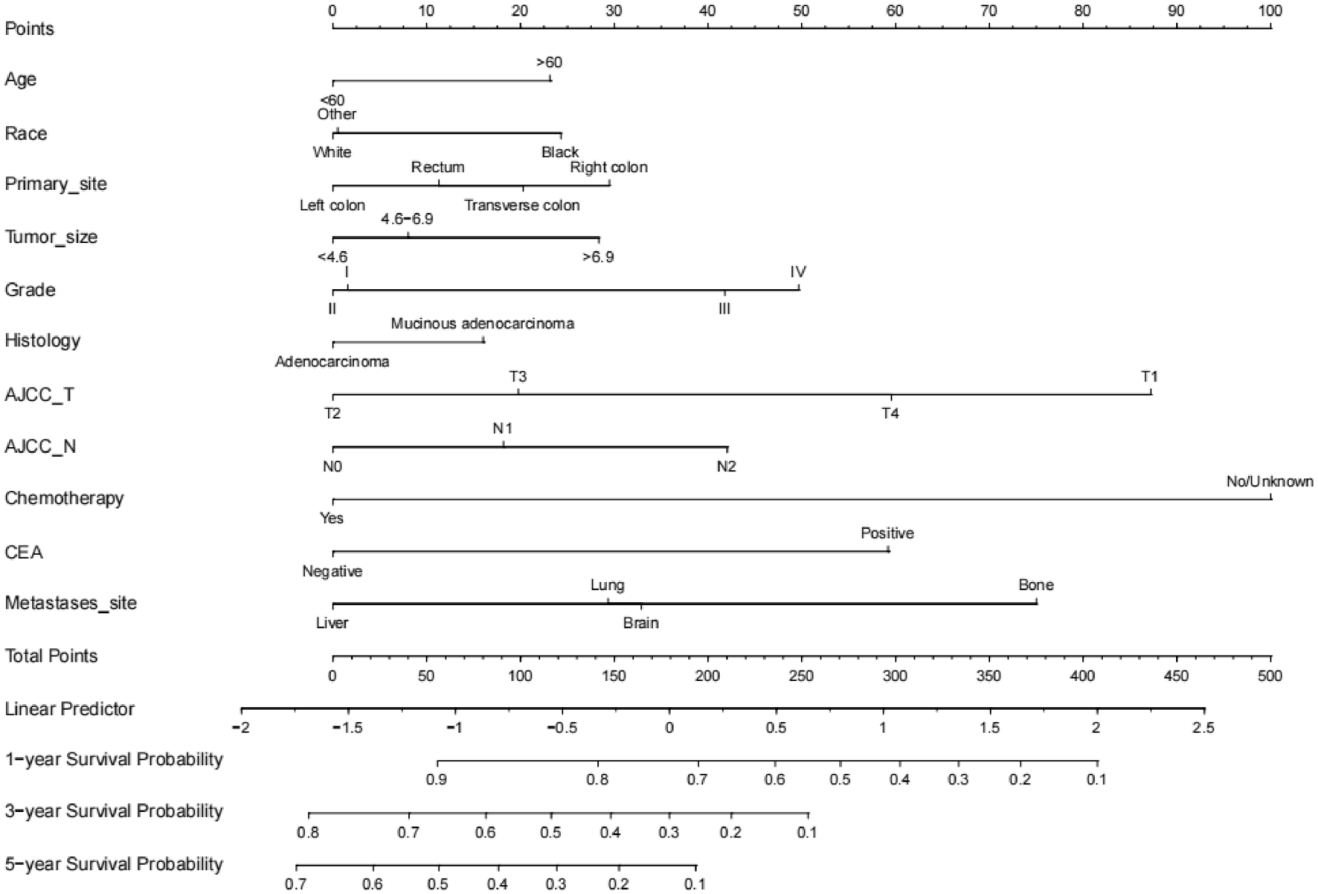
Nomogram predicting 1-, 3-, and 5-year OS rates of mCRC patients

Based on the selected independent prognostic factors affecting patients’ OS, we constructed nomogram model to predict patient OS (Fig. 4). In patients with mCRC, nomogram predicted OS at 1, 3, and 5 years. All factors were given a score between 0 and 100 based on the amount they contributed to the nomogram. The scores for each category were added to create a final score for each patient, with chemotherapy, T stage, and metastases site showing the greatest effects on prognosis.

### Verification of the nomogram prediction model

**Fig. 5:**
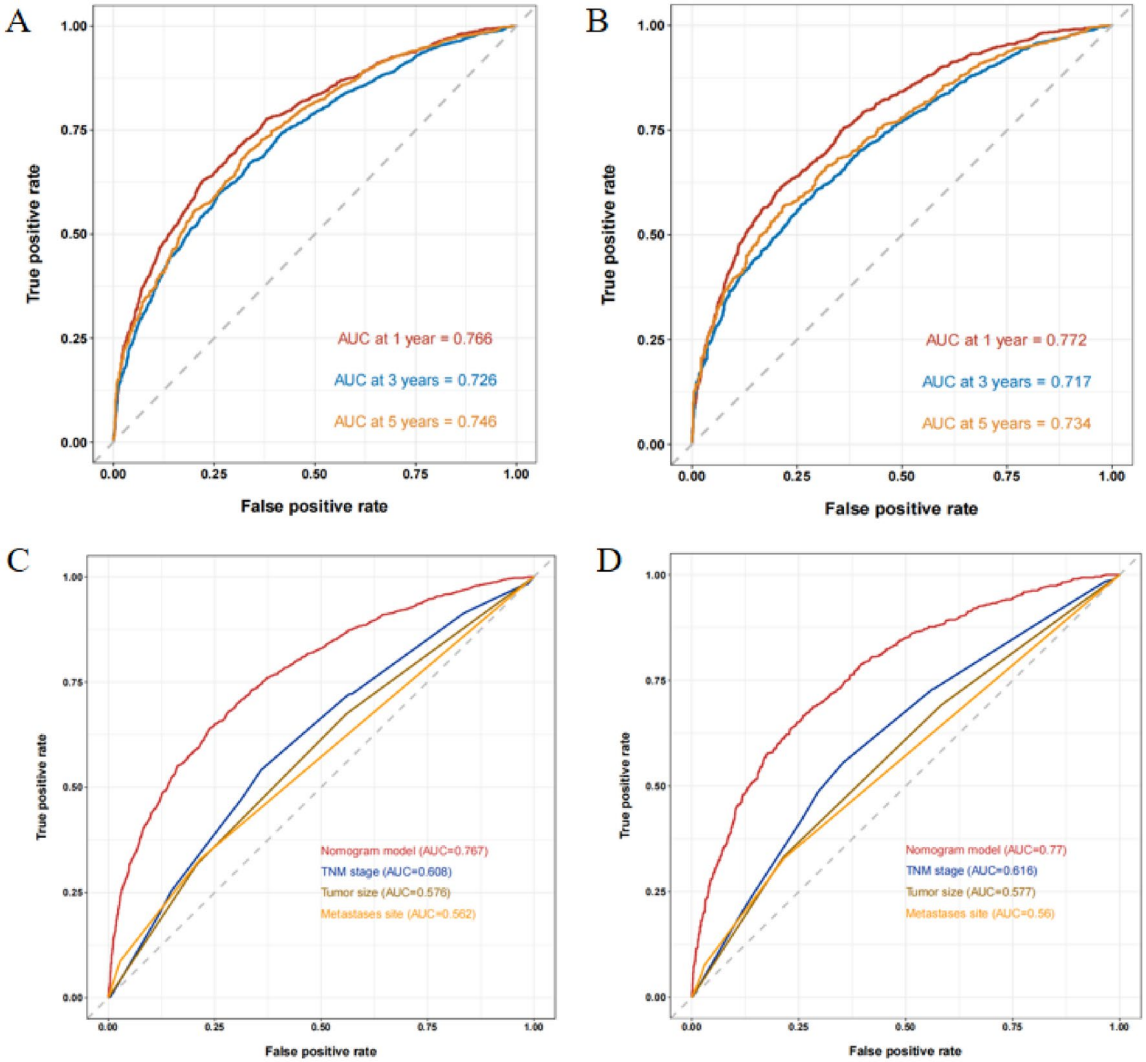
ROC curves and AUC values for 1-, 3- and 5-year overall survival predictions in training cohort (**A**) and validation cohort (**B**); ROC curve of training cohort (**C**) and validation cohort (**D**) compared between the prognostic model and TNM staging model and other prognostic factors in predicting OS

**Fig. 6:**
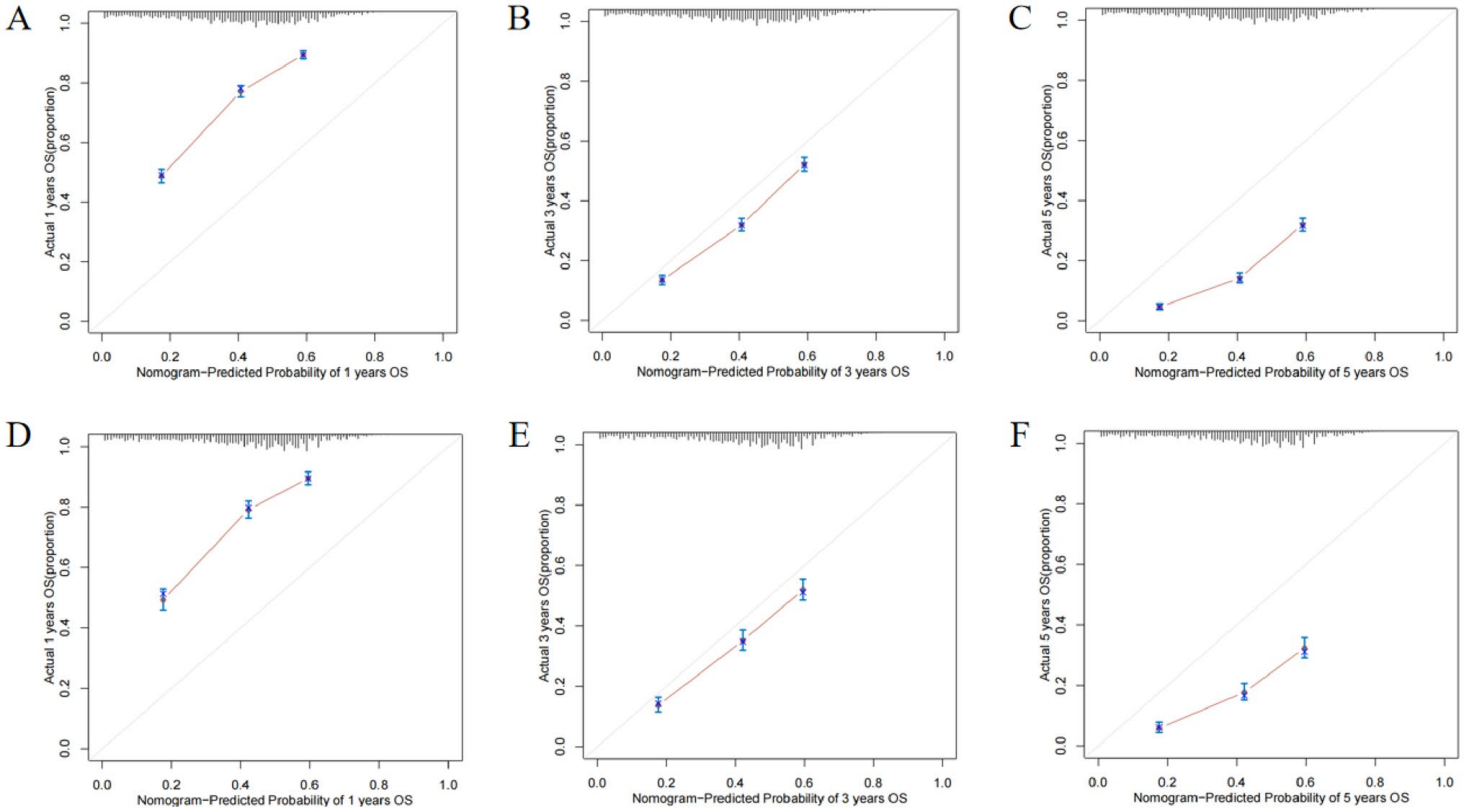
Calibration plots of the nomogram describing 1-, 3- and 5-year OS in training cohort (**A**–**C**) and validation cohort (**D**–**F**)

ROC curve of training cohort’s nomogram was drawn to predict the OS of patients at 1, 3, and 5 years, and then calculated their AUC values to be 0.766, 0.726, and 0.746 (Fig. 5A). At the same time, the AUC values of validation cohort at 1, 3, and 5 years were 0.772, 0.717, and 0.734 (Fig. 5B). The TNM staging-based ROC curve was then built in training cohort and validation cohort, and the AUC values for the 1-year period were 0.608 (Fig. 5C) and 0.616 (Fig. 5D), respectively. These result showed that the prediction model developed in this study was advantageous to the model based on the TNM staging system. In addition, the calibration diagrams for the two cohorts’ prediction curve and ideal curve fit together well, indicating the model’s high degree of accuracy (Fig. 6).

### Decision curve analysis

**Fig. 7:**
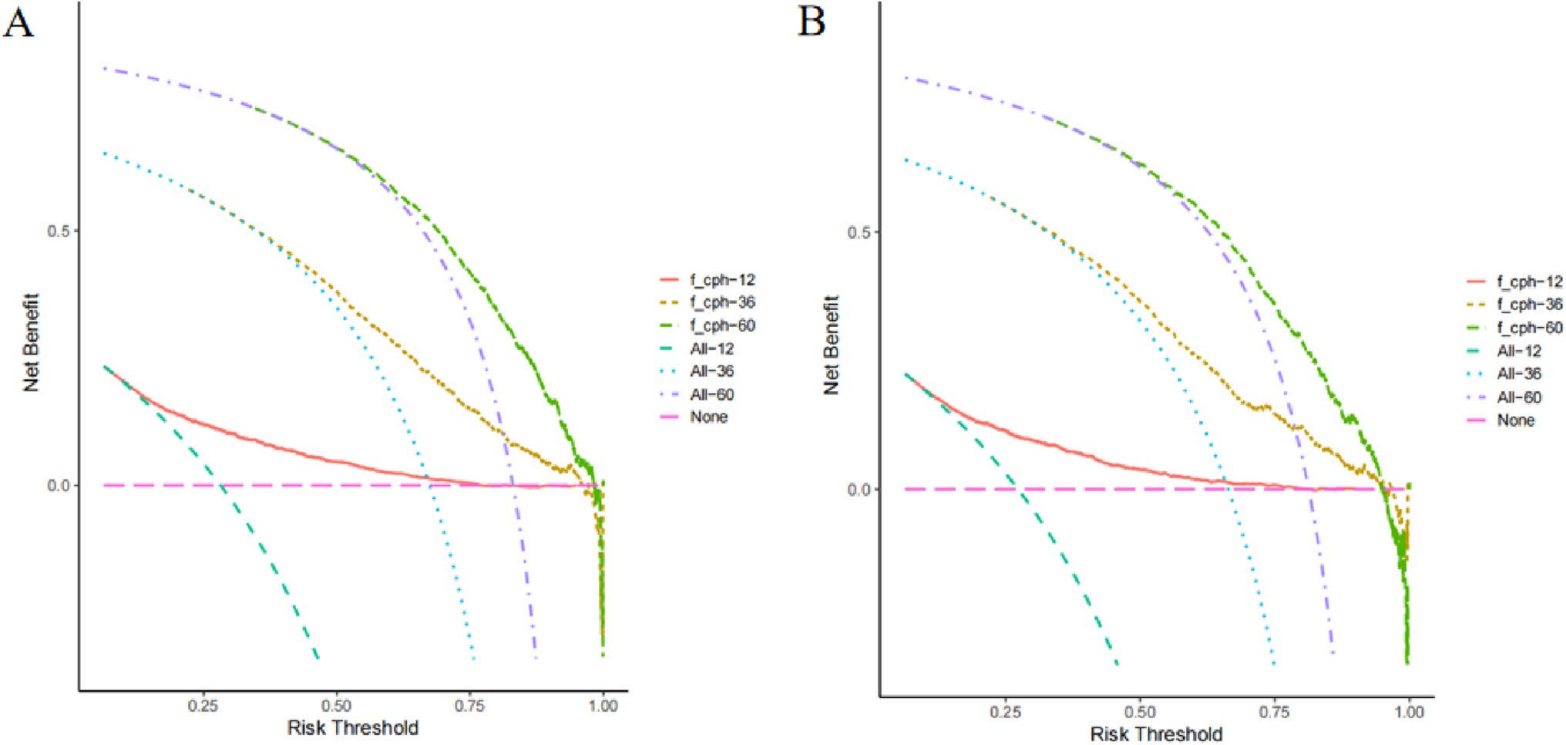
The decision curve analysis (DCA) of nomogram for predicting patients’ OS at 1, 3, and 5 years in training cohort (**A**) and validation cohort (**B**)

Decision curve analysis (DCA) was performed at 1, 3 and 5 years of OS in training cohort (Fig. 7A) and validation cohort (Fig. 7B). In both data cohorts, the nomogram demonstrated good clinical value, with a greater clinical utility value in predicting OS at 3 and 5 years and only moderately less at 1-year OS.

## Discussion

CRC is one of the most common malignant tumors globally, accounting for 10% of newly diagnosed cases of malignancy [1]. The TNM staging system proposed by AJCC is based on the depth of local invasion, without taking into consideration the size of the primary tumor. Some studies have suggested that tumor size may be a useful supplement to the TNM staging system to enhance the accuracy of prognostication for CRC [12–16]. Compared to invasion depth, the tumor size can be obtained via imaging examinations, which are simple, safe, non-invasive and accurate. However, there is no unified optimal cutoff value to stratify tumor size. Shiraishi et al. [17] analyzed 95 patients with T4 stage CRC and determined the optimal threshold of 5.0 cm for tumor size via ROC curves, confirming that tumor size was significantly associated with prognosis. Deng et al. [18] analyzed clinical pathological parameters of 1250 colorectal cancer hepatopulmonary metastases (CRCHPM) patients and constructed a reliable model with 7 independent prognostic factors. The tumor size threshold selected in the study was 5.5 cm and it was observed that the larger the tumor, the poorer the prognosis and the greater the risk of liver and lung metastasis. Saha et al. [19] conducted a subgroup analysis of 300,386 CRC patients with thresholds of 2.0, 4.0, and 6.0 cm, and found that the 5-year survival rate decreased significantly with the increase in tumor size, and this trend was observed regardless of lymph node metastasis. Another study that stratified the metastatic potential and risk of disease recurrence of 1538 CRC patients showed that tumor size exceeding 5.0 cm was associated with an increased rate of disease recurrence [20]. Our study results confirmed previous findings, although in our overall study population, tumor size had less significant impact on prognosis compared to TNM staging, significant findings were observed across different AJCC stages. Compared to smaller primary tumors, those over 6.9 cm in mCRC patients were associated with worse survival rates independent of other variables. One possible explanation for the negative effect of tumor size on T stage may be the inaccuracy of tumor size calculation. It is conceivable that, for tumors with advanced stages, accurately measuring tumor size is difficult as the invasion extent of the bowel wall may be larger than the maximal diameter of the cancerous extent on the mucosa. Thus, the maximal horizontal diameter may not accurately reflect the extent of tumor growth with advanced infiltration. On the other hand, for tumors confined to the submucosa, growth into the lumen may be the main pattern, and the tumor size measured at this stage may be the dominant index that depicts the growth of tumor. Nevertheless, we believe there are other potential causes for the impact of tumor size that need further investigation.

The distant metastasis of tumors, including CRC, is thought to comprise a multistep process of three phases: (1) Invasion phase: in situ tumor cells increase their invasiveness through epithelial–mesenchymal transition (EMT) processes, penetrating the surrounding tissues and migrating to the blood vessels or lymphatic vessels, creating circulating tumor cells (CTCs); (2) circulation phase: platelets adhere directly to the surface of CTCs, creating micro-thrombi structures that reduce the recognition and clearance of the immune system; (3) settlement phase: CTCs settle in distant organs, forming a pre-metastatic niche, a microenvironment characterized by immune suppression, enabled by the secretion of cytokines or exosomes from the primary tumor site [21]. The classical metastasis model suggests that distant metastasis may take place with the development of time and/or tumor size increase [22], which is in accord with the insights from this study that primary tumors in the colon or rectum kept growing, becoming more invasive, accumulating damaging mutations and eventually gaining the capacity for distant metastasis, leading to mCRC. Some have postulated that, similar to breast cancer, mCRC belongs to an early dissemination model [23], which we refer to as the “genius tumor”, as these tumors possess metastasis-related mutant alleles at an early stage. However, due to the lack of multi-omics molecular characterization of CRC in this study, it was impossible to distinguish between patients belonging to the growth-accumulation model and those to the early dissemination model. We also observed that the larger the tumor size of bone or brain metastatic patients, the poorer the prognosis, though the *P* value was over 0.05. This might be due to the fact that these patients have a poor prognosis themselves, for example, the prognosis of brain metastatic patients is a median survival time of 3–6 months upon diagnosis and no effective therapy [24].

The relationship between tumor size and prognosis of CRC patients has long been of great concern to clinicians. This study, based on a large-scale clinical data analysis, found that tumor size is an independent prognostic factor for mCRC patients. Nevertheless, certain limitations of the study could not be overlooked. Despite the large size of SEER database, our stratification by tumor size and metastatic site resulted in relatively small subgroups, reducing statistical power to detect small differences. This may help explain why we failed to detect significant associations between tumor size and metastatic site in the subgroups of bone and brain metastatic patients. In addition, information on missing molecular characteristics such as BRAF mutation status, microsatellite status, adjuvant therapy, or pathology techniques was not included in the SEER database. Furthermore, although this study was conducted in large populations, the proportion of Asian patients was still too small, thus necessitating further multicentral research to validate the results of this study.

## Conclusion

Our findings suggested that patients with mCRC who had lung or liver metastases have a poorer prognosis when their primary tumors are larger. We developed an accurate prognostic risk assessment model for such patients, allowing them to estimate their overall score via a nomogram and calculate their chances of survival. This plays a significant role in providing clinical guidance. Further prospective research is necessary to determine the role of tumor size in clinical staging models for management selection.

## Data Availability

Publicly available datasets were analyzed in this study. These data can be found here: https://seer.cancer.gov/seerstat/.
